# Factors associated with the willingness of young people in higher education to adhere to syphilis screening

**DOI:** 10.1590/0034-7167-2024-0423

**Published:** 2025-09-01

**Authors:** Ana Paula Ferreira Holzmann, Ana Paula Forte Camarneiro, Aliete Cunha-Oliveira, João Luíz Grandi, Cristiano Leonardo de Oliveira Dias, Janer Aparecida Silveira Soares, Yan Lucas Martins Silva, Mônica Taminato, Dulce Aparecida Barbosa

**Affiliations:** I Universidade Estadual de Montes Claros. Montes Claros, Minas Gerais, Brazil; II Escola Superior de Enfermagem de Coimbra. Coimbra, Portugal; III Universidade Federal de São Paulo. São Paulo, São Paulo, Brazil

**Keywords:** Syphilis, Serologic Tests for Syphilis, Health Knowledge, Attitudes, Practice, Adults, Young, Graduate Education., Sífilis, Serodiagnóstico de la Sífilis, Conocimientos, Actitudes y Práctica en Salud, Adulto Joven, Enseñanza Superior.

## Abstract

**Objectives:**

to investigate factors associated with willingness to know one’s syphilis serological status among higher education students.

**Methods:**

a cross-sectional and analytical study conducted with higher education students in Portugal. Data collection was carried out through an online questionnaire. Data were analyzed by Poisson logistic regression with robust variance, using SPSS-24.

**Results:**

413 students were included, most of whom were female, with an average age of 20.5 years and enrolled in health courses. Behavioral vulnerabilities were observed, such as non-use of condoms and use of alcohol/other drugs, low risk perception, and poor knowledge about syphilis. Less than half of students were willing to take the syphilis test.

**Conclusions:**

adherence to syphilis screening was low and associated with uncommon factors, such as less knowledge, low risk perception, younger age, number of sexual partners and attending courses in areas other than health.

## RESUMEN

**Objetivos:**

investigar los factores asociados con la disposición a conocer el estado serológico de sífilis en estudiantes de educación superior.

**Métodos:**

estudio transversal y analítico, realizado con estudiantes de educación superior en Portugal. La recogida de datos se realizó a través de un cuestionario en línea. Los datos se analizaron mediante regresión logística de Poisson con varianza robusta, utilizando SPSS-24.

**Resultados:**

se incluyeron 413 estudiantes, la mayoría del sexo femenino, con edad promedio de 20,5 años y matriculados en carreras de salud. Se observaron vulnerabilidades conductuales, como no uso de condones y consumo de alcohol/otras drogas, baja percepción de riesgo y escaso conocimiento sobre la sífilis. Menos de la mitad de los estudiantes estaban dispuestos a hacerse la prueba de sífilis.

**Conclusiones:**

la adherencia al tamizaje de sífilis fue baja y se asoció con factores inusuales, como menor conocimiento, baja percepción de riesgo, edad más joven, número de parejas sexuales y asistencia a cursos en áreas distintas a la salud.

## INTRODUCTION

Sexually transmitted infections (STIs) have maintained their historical mark and, in the 21^st^ century, continue to challenge public health globally, disproportionately affecting young people and negatively impacting the quality of life of those affected and their families. It is estimated that, worldwide, one million new cases of STIs occur every day and that, of these, 374 million are curable infections, such as syphilis (7.1 million), chlamydia (129 million), gonorrhea (82 million) and trichomoniasis (156 million)^(1)^.

Among STIs, syphilis has stood out in the global epidemiological scenario, with an increase in cases in the young adult population. Although it has an established, low-cost and easy-to-implement treatment, the disease has high morbidity and causes a series of complications, which can evolve into chronicity, in addition to facilitating contamination by the Human Immunodeficiency Virus (HIV)^(2-4)^.

In 2016, 29,365 cases of syphilis were recorded in the European Union (incidence: 6.1/100,000), and in 2019, this number rose to 35,039 new cases (incidence: 7.4/100,000). Portugal is one of the countries with the highest incidence of cases in recent years, with almost 50% of cases in the population aged between 15 and 35 years. In the country, syphilis cases increased from 43 reported cases in 2015 (0.4 cases per 100,000 inhabitants) to 419 cases in 2019 (4.1 cases per 100,000 inhabitants), representing an increase of 874.42% in the number of notifications in the country^(4)^.

The vulnerability of young people to syphilis and other STIs is diverse, involving aspects such as early sexual initiation, frequent change of sexual partners and irregular use of condoms. Added to these are the need for acceptance and inclusion in social groups, and the consumption of alcohol and other drugs^(4-6)^. Entering higher education appears to contribute to increasing young people’s vulnerability to STIs. University life gives young people more autonomy and freedom to expand their circle of friends and experience new things, such as alcohol and drug use and unsafe sex, which were previously limited or inhibited by greater family closeness and control^(7)^.

One of the main factors that make early diagnosis of syphilis difficult is the long latency period of the infection. During this phase, in which people remain asymptomatic, there is usually no motivation to undergo testing, which goes against the World Health Organization (WHO) recommendation that all exposed people undergo testing for STIs. Early diagnosis has a positive impact, as it allows for the institution of appropriate treatment, the reduction of transmission between partners and long-term complications, especially on the reproductive health of young people, in addition to preventing new cases of congenital syphilis^(1,8)^.

The prevention and control of STIs, especially HIV infection, syphilis and hepatitis B and C, are interconnected with the promotion of health, gender equality, education and the reduction of inequalities, and are therefore fundamental to achieving the United Nations (UN) 2030 Agenda Sustainable Development Goals (SDGs), which seeks to respond to the main global challenges of the 21^st^ century, such as improving individual health, sexual health and the well-being of all people^(1)^. Among the strategies to expand and anticipate the diagnosis of these infections, the most important are encouraging and mobilizing individuals to undergo voluntary testing, especially for more vulnerable populations, such as adolescents and young people, and preferably through rapid tests (RTs)^(4)^.

Despite the increase in reported cases of STIs, there is a lack of studies investigating the epidemiology of these infections in Portugal, especially among young people. Given that the university environment can foster risk behaviors that increase students’ vulnerability, research that explores and analyzes these behaviors becomes essential to support the development of public policies and health promotion programs aimed at this population.

## OBJECTIVES

To investigate the factors associated with the willingness of higher education students to know their syphilis serological status.

## METHODS

### Ethical aspects

This study is an excerpt from a post-doctoral research project entitled *“Perfil epidemiológico, conhecimento e práticas comportamentais relacionadas às infecções de transmissão sexual entre estudantes universitários*”. It was conducted in accordance with national and international ethics guidelines, approved by the Research Ethics Committees of the *Escola Superior de Enfermagem de Coimbra*, in Portugal, and the *Universidade Federal de São Paulo*, in Brazil, whose opinion is attached to this submission.

### Study design, location and period

This is a cross-sectional descriptive-correlational epidemiological study, guided by the STrengthening the Reporting of OBservational studies in Epidemiology checklist. It was carried out in the city of Coimbra, central region of Portugal, with data from two higher education institutions, from October 2022 to January 2023.

### Sample; inclusion and exclusion criteria

The initial sample involved 599 students from various areas of knowledge, selected by convenience. Students between 18 and 25 years old, regularly enrolled in one of the courses offered by institutions and having initiated sexual life, resulting in a final sample of 413 students, were included.

### Study protocol

Data collection was carried out through an online questionnaire, sent to students via Google Forms^®^, by the higher education institutions participating in the study. The Informed Consent Form was obtained from all individuals involved in the study before accessing the questionnaire.

The instrument addressed questions about sociodemographic data, knowledge, behaviors and attitudes regarding STIs, in addition to a question about students’ interest in undergoing RT for syphilis, considered as a dependent variable in this section. The questionnaire content validity was carried out by three experts in the field, two from Portugal and one from Brazil. Moreover, the instrument was submitted to a pre-test with a class of 20 university students, who were not included in this study.

To assess knowledge about syphilis, five questions were asked. The answers to these questions were categorized as true or false/do not know. Knowledge was classified as “good” for those who answered four to five questions correctly, “regular” for those who answered three questions correctly, and “poor” for those who answered zero to two questions correctly.

The research data, generated in a Microsoft Excel^®^ spreadsheet by Google Forms^®^, were transferred to a database created in the Statistical Package for the Social Sciences version 24 for Windows, where variables were organized and, when necessary, recategorized.

### Analysis of results, and statistics

To characterize the sample, descriptive statistics were performed using the distribution of absolute and relative frequencies, means and standard deviation (SD). The magnitude of the association between the dependent variable and the other variables investigated was estimated using the Prevalence Ratio, using the Poisson logistic regression model with robust variance.

Initially, bivariate analyses were performed using Pearson’s chi-square test and Fisher’s exact test, when appropriate, for categorical variables and Student’s t-test (independent samples) for numerical variables.

Finally, variables that presented a descriptive level (p-value up to 0.20) were selected for the multiple model, whose significance level was set at 0.05. The final multivariate model was constructed using the backward method, which removes factors with a p-value > 0.05 one by one from the model.

## RESULTS

The sample consisted mostly of young women (79.9%), aged between 18 and 25 years, self-classified as white (93.7%), single (97.6%), Catholic (67.7%) and mainly from the city of Coimbra (34.9%). As for the course attended, the health area stood out (45.3%) in relation to the other areas, and more than half of the sample (53.7%) was enrolled in the final years of their training (Table 1).

**Table 1:** Distribution of sociodemographic variables and association with willingness to undergo syphilis screening using rapid testing, Coimbra, Portugal, 2022-2023

Variables			Adherence to syphilis screening				*p* value
			Yes		No		
	n^*^	%	n	%	n	%	
Sex							
Female	330	79.9	97	30.5	221	69.5	0.457
Male	83	20.1	21	26.3	59	73.8	
Age in years (M±SD)	20.50 ±1.73						
18-19	119	28.8	27	23.5	88	76.5	0.086
20-25	294	71.2	91	32.2	192	67.8	
Skin color							
White	387	93.7	109	29.3	263	70.7	0.566
Non-white	26	6.3	09	34.6	17	65.4	
Marital status							
Married/stable union	10	2.4	04	40.0	06	60.0	0.493 (F)
Single	401	97.6	114	29.5	272	70.5	
Origin							
Coimbra	144	34.9	38	27.5	100	72.5	0.215
Other cities Portugal	255	61.7	73	29.7	173	70.3	
Other countries	14	3.4	07	50.0	07	50.0	
Course area							
Health	187	45.3	82	45.3	99	54.7	< 0.001
Other areas	226	54.7	36	16.6	181	83.4	
Course grade							
1^st^ and 2^nd^ grades	191	46.2	49	26.6	135	73.4	0.222
3^rd^ grade or more	222	53.8	69	32.2	145	67.8	

M - mean SD - standard deviation;

* Values with sums other than 413 refer to missing data.

Sociodemographic variables that were associated with the outcome (p< 0.20) and that were included in the Poisson logistic regression model were age, religion and area of the course attended (Table 1).

Concerning knowledge about syphilis (Table 2), the mean number of correct answers among the five questions was 2.33 (SD = 1.54). The question with the highest frequency of correct answers was “Untreated syphilis can cause neurological and cardiovascular problems” (57.7%), and the one with the highest frequency of errors was “Spots on the body and hair loss can be signs of syphilis” (31.5%).

**Table 2:** Knowledge about syphilis among higher education students, Coimbra, Portugal, 2022-2023

Questions assessed	n^*^	%
Untreated syphilis can cause neurological and cardiovascular problems		
Correct answer	240	58.3
Incorrect answer/does not know	172	41.7
Syphilis is a sexually transmitted infection with no cure, but it can be prevented through vaccination		
Correct answer	204	49.5
Incorrect answer/does not know	208	50.5
Syphilis can be transmitted through the use of toilets, swimming pools, park benches		
Correct answer	187	45.4
Incorrect answer/does not know	225	54.6
Spots on the body and hair loss can be signs of syphilis		
Correct answer	136	32.9
Incorrect answer/does not know	277	67.1
Syphilis during pregnancy can cause miscarriage, premature birth and the birth of children with serious health problems		
Correct answer	213	51.8
Incorrect answer/does not know	198	48.2
Average number of hits (M ± SD)	2.33 ±1.54	

M - mean; SD - standard deviation;

* Values with sums other than 413 refer to missing data

In the overall classification, the poor level of knowledge about the questions assessed prevailed (Table 3). Table 3 presents the results related to the behavioral variables of students investigated, knowledge about syphilis and the association between these variables and willingness to undergo screening through RT.

**Table 3:** Behavioral characteristics and knowledge about syphilis among higher education students and association with willingness to undergo syphilis screening using rapid testing, Coimbra, Portugal, 2022-2023

Variables			Willingness to take the rapid test for syphilis				*p* value
			Yes		No		
	n^*^	%	n	%	n	%	
Sexual orientation							
Heterosexual	330	84.2	91	28.5	228	71.5	0.076
Homosexual/bisexual	62	15.8	24	40.0	36	60.0	
Age of sexarche (M ± SD)	16.73 ± 1.71						
Over 18 years	48	12.6	13	27.1	35	72.9	0.340
16-18 years	247	64.8	68	28.5	171	71.5	
15 years or younger	86	22.6	31	36.5	54	63.5	
Steady partner in the last 6 months							
No	92	22.3	22	23.9	70	76.1	0.262
Yes	321	77.7	96	29.9	225	70.1	
Casual partner in the last 6 months							
No	231	55.9	60	26.0	171	74	0.188
Yes	182	44.1	58	31.9	124	68.1	
Number of steady sexual partnerships^**^ (M ± SD)	0.86 ± 0.56						
None	62	15.0	15	25.4	44	74.6	0.676
One partner	289	70.0	84	29.9	197	70.1	
Two or more partners	62	15.0	19	32.8	39	67.2	
Number of casual sexual partnerships ^**^ (M ± SD)	0.90 ± 1.65						
None	192	51.3	52	28.0	134	72.0	0.437
One or two partners	141	37.7	44	32.1	93	67.9	
Three or more partners	41	11.0	15	37.5	25	62.5	
Total number of sexual partnerships^**^ (M ± SD)	0.90 ± 1.65						
Up to 2 partners	347	84.0	92	26.5	255	73.5	0.034
3 or more partners	66	16.0	26	39.4	40	60.6	
Use of condoms during the first sexual intercourse							
Sim	287	69.5	82	29.7	194	70.3	0.968
No/does not remember	126	30.5	36	29.5	86	70.5	
Used condom during vaginal/anal sex^**^							
Yes/most of the time	171	49.1	47	27.5	124	72.5	0.283
No/minority of the time	177	50.9	58	32.8	119	67.2	
Used condom during oral sex^**^							
Yes/most of the time	69	18.2	16	23.2	53	76.8	0.188
No/minority of the time	311	81.8	97	31.2	214	68.8	
Previous or current history of sexually transmitted infections							
No/does not know	387	93.7	112	28.9	275	71.1	0.005
Yes	26	6.3	6	23.1	20	76.9	
Use of psychoactive substances^**^							
No	34	8.2	15	44.1	19	55.9	0.036
Use/already used	379	91.8	103	27.2	276	72.8	
Perceived risk of contracting sexually transmitted infections							
Yes	141	34.1	54	38.3	87	61.7	0.002
No/does not remember	272	65.9	64	23.5	208	76.5	
Has been tested for syphilis before							
Yes	40	10.0	15	37.5	25	62.5	0.252
No/does not remember	358	90.0	103	28.8	255	71.2	
Knowledge about syphilis							
Good	101	24.5	52	53.1	46	46.9	< 0.001
Regular	91	22.0	25	28.7	62	71.3	
Poor	221	53.5	41	19.2	172	80.8	

* Values with sums other than 413 refer to missing data;

** Information relating to the last 6 months.

From bivariate analysis (Table 3), the following variables were selected to compose the Poisson logistic regression model (p < 0.20): sexual orientation; had a casual/occasional partner in the last 6 months; total number of sexual partners in the last 6 months (steady + casual); use of condoms during oral sex in the last 6 months; use of psychoactive substances (alcohol and/or other drugs); perceived risk of contracting an STI at some point in life; and knowledge about syphilis.

As for the numerical variables, those that presented a significance level of up to 0.20 were selected by t-test for independent samples, such as age in years (p=0.018), number of steady partners in the last 6 months (p=0.160) and total number of sexual partners in the last 6 months (p=0.151) (data not shown in table).

After adjustment, the final logistic model was composed of the variables (p< 0.05): as course area, knowledge about syphilis, perception of risk for STI, total number of sexual partners in the last 6 months and age in years (Table 4).

**Table 4:** Prevalence Ratio of variables associated with adherence to syphilis screening among higher education students, Coimbra, Portugal, 2022-2023 (logistic model estimates)

Independent variables	Adjusted PR	95% CI	p value^*^
Course area			
Health	1		
Other areas	1.158	1.094-1.225	<0.001
Knowledge about syphilis			
Good	1		
Regular	1.152	1.069-1.240	<0.001
Poor	1.137	1.045-1.236	0.003
Perception of risk for sexually transmitted infections			
Yes	1		
No	1.069	1.011-1.130	0.019
Total number of sexual partners in the last 6 months			
Up to 2 partners	1		
3 or more partners	0.916	0.850-0.987	0.008
Age in years	0.980	0.966-0.995	0.021

PR - Prevalence Ratio; CI - Confidence Interval;

* Pearson’s chi-square test.

## DISCUSSION

In general terms, the socio-behavioral profile of higher education students found in this study is similar to that described in other national and international studies^(9-13)^, i.e., it is mostly made up of young, single female students who engage in riskier behaviors, such as low adherence to condoms, use of alcohol and other drugs.

These characteristics are considered markers of greater vulnerability to STIs and, when associated with university environments, place higher education students as a population that requires greater attention and visibility in public health policies^(9,11,14)^.

Considering syphilis an infection that has serious consequences for human health, especially when it affects the fetus, it is understood that its prevention is urgent and necessary, especially in the population of reproductive age. Thus, as with other STIs, global health authorities recommend, in addition to the use of condoms, alternative prevention methods, including periodic testing for STIs^(1,2,4)^.

For this purpose, RTs are considered a good strategy, as they provide the result in up to 30 minutes, which allows measures to be taken immediately in the event of reactive results^(2)^. Thus, RT is another example of public action, with the objective of controlling STIs, which becomes more effective the greater the participation of citizens in the form of voluntary adherence to the procedure^(15)^.

However, studies^(4-6,10,13,16,17)^ have shown low interest and adherence to STI screening among young people, which is supported by the present study, in which less than 30% of students expressed interest in rapid testing for syphilis. This leads an individual to accept or reject an invitation to take a screening test, and can be determined by several factors; among them, the self-perception of risk/vulnerability in the face of the situation or problem in question stands out.

The perception of subjective risk is one of the dimensions proposed by the Health Belief Model (HCM), initially developed by Rosenstock in the mid-20^th^ century. This dimension is called perceived susceptibility, one of the predictors of the proposed preventive health behaviors. Perceived susceptibility varies from person to person and refers to the subjective risk that a person has of contracting a disease^(18)^.

This theoretical model was proven by studies that identified the absence or low perception of risk as the main obstacle to adherence to preventive behaviors, including carrying out diagnostic tests for STIs^(3,19,20)^. In fact, in this study, a high frequency of students (65.9%) did not perceive themselves to be at risk of acquiring STIs; however, contradictorily, multivariate analysis revealed that it was precisely these students who adhered, in a more significant manner, to syphilis screening, possibly motivated by curiosity.

This finding demonstrates, as predicted by HBM, that the perception of risk alone does not define what individuals’ action will be and that other dimensions must be considered, such as: perceived severity, i.e., the perception that individuals have about the impact that the event may have on their lives; the perceived benefits that such preventive actions and changes in habits will provide; and the barriers, difficulties or other aspects perceived as negative related to the action^(18)^.

Thus, another hypothesis for low adherence to the test found in this study would be the lack of perception regarding the severity of syphilis, which can be inferred from the low knowledge of students investigated in relation to the infection or the fact that it is a curable infection, which reduces the concern about its transmission^(3)^.

Finally, the barriers perceived by students may outweigh the benefits and contribute to non-adherence to screening, even among those who are aware of their vulnerability and the severity of the infection. Negative feelings, linked to stereotypes about behavior and social morality that still surround the universe of STIs today, such as fear, shame and embarrassment, may better explain low adherence to screening, as already demonstrated by other studies^(21,22)^.

Another unexpected finding in this study was the significantly higher prevalence of screening adherence among students who had fewer sexual partners in the last semester. In fact, the opposite would be expected, since changing sexual partners more frequently leaves people more exposed to STIs, which usually drives the search for testing.

However, vulnerable people do not always perceive themselves to be at risk and, among those who do, the attitude of not being tested may be anchored in the previous hypothesis: negative feelings as barriers to testing. Moreover, people may not consider being tested for STIs because they appear to be healthy^(23)^.

Although studies have demonstrated a positive association between knowledge and the intention or performance of screening test for STIs^(14,23,24)^, in the present study, the association found was inverse, i.e., the intention to perform the test was more prevalent among students who presented less knowledge about syphilis. It was also expected that students in the health field, who theoretically have greater knowledge about STIs, would adhere more significantly to screening, which also did not occur.

These findings corroborate and reinforce the hypothesis that knowledge, although fundamental, is not always sufficient for the adoption of preventive measures, as there are intervening factors, related to social and cultural norms, pressures exerted by the group and the social network itself, which can overlap and lead to inappropriate behavior, observed in this and other studies^(7,8,14,21)^, such as irregular condom use, frequent change of sexual partners, use of alcohol and other drugs, and low performance or willingness to perform diagnostic screenings.

Finally, it was found that age influenced the decision to perform RT for syphilis, demonstrating that for each year of increase in age there was a 2% decrease in the prevalence of students intending to adhere to screening. This result is contrary to what is expected, since, as people mature, they tend to have greater awareness of the risks already experienced and, therefore, greater motivation to perform diagnostic screenings^(17)^.

However, as already discussed, students’ risk perception, in the present study, proved to be a weak predictor of prevention, negatively associated with the willingness to undergo screening, which certainly does not explain the attitude of these young people.

The variables defined by the multiple model, which best explained the willingness to perform RT for syphilis, in this study, are at least surprising and lead us to reflect on how they establish a relationship between themselves and arrive at the same hypothesis: that the perception of risk, common among people with a greater number of sexual partners, who have greater knowledge, who attend health courses and who are older, can work, contrary to what most studies indicate, which is a barrier to carrying out diagnostic screenings.

Considering that the decision about whether to take the test can be complex, it is believed that promoting professional advice on the risks of infection and awareness about the benefits of taking the test could help in the decision to adhere to screening, which, unfortunately, was not possible due to the method chosen for data collection in this study. In a study that investigated the factors associated with late HIV diagnosis^(20)^, 41.4% of respondents decided to be tested only after receiving advice from a healthcare professional, thus demonstrating the importance of counseling in the context of STIs.

### Study limitations

This study has some limitations, such as the use of a sample selected for convenience and limited to a Portuguese geographic region, which may not linearly reflect the reality of the country. Furthermore, due to the design used, it is not possible to establish a temporal and causal relationship between the willingness to perform RT for syphilis and the other variables analyzed.

### Contributions to education, health and nursing

Despite the limitations, it is believed that the results presented may encourage reflection on the need for curricular adaptations and the development of research and extension programs/projects in higher education institutions, aiming to increase health literacy and promote students’ sexual health. Furthermore, it is expected that these findings will encourage the implementation of comprehensive institutional student health policies that not only address sexual health, but also other relevant aspects, consolidating the role of universities as health promoters. In this context, the importance of the inclusion of nurses, professionals with technical competence to strengthen health promotion within higher education institutions, is highlighted.

## CONCLUSIONS

Adherence to syphilis screening tests among students was low and was associated with atypical factors, such as lower level of knowledge, low perception of risk, younger age, fewer sexual partnerships and participation in courses in areas unrelated to health.

These results invite us to reflect on the different psychosocial factors that hinder STI testing and the importance of considering them in discussions and planning interventions aimed at this target audience. In this context, it is essential that higher education institutions recognize themselves as appropriate spaces for promoting the sexual and reproductive health of their students, in line with WHO guidelines and the SDGs.

## Data Availability

*https://doi.org/10.1590/SciELOPreprints.9513.*
