# Supplementary material for: Factors associated with the willingness of young people in higher education to adhere to syphilis screening
Source: Rev Bras Enferm. 2025 Sep 1;78(Suppl 1):e20240423. doi: 10.1590/0034-7167-2024-0423 (PMC12407882; doi:10.1590/0034-7167-2024-0423)
Supplement: Translation

# Translation: Fatores associados à disposição de jovens do ensino superior para adesão ao rastreio da sífilis

**DOI:** 10.1590/0034-7167-2024-0423pt

**Authors:** Ana Paula Ferreira Holzmann, Ana Paula Forte Camarneiro, Aliete Cunha-Oliveira, João Luíz Grandi, Cristiano Leonardo de Oliveira Dias, Janer Aparecida Silveira Soares, Yan Lucas Martins Silva, Mônica Taminato, Dulce Aparecida Barbosa

**Affiliations:** I Universidade Estadual de Montes Claros. Montes Claros, Minas Gerais, Brasil; II Escola Superior de Enfermagem de Coimbra. Coimbra, Portugal; III Universidade Federal de São Paulo. São Paulo, São Paulo, Brasil

**Keywords:** Sífilis, Sorodiagnóstico da Sífilis, Conhecimentos, Atitudes e Prática em Saúde, Adultos Jovens, Ensino Superior

## Abstract

**Objetivos:**

investigar os fatores associados à disposição para conhecer o estado sorológico para sífilis entre estudantes do ensino superior.

**Métodos:**

estudo transversal e analítico, realizado com estudantes do ensino superior de Portugal. A coleta de dados ocorreu por meio de questionário *online*. Os dados foram analisados pela regressão logística de Poisson com variância robusta, utilizando o SPSS-24.

**Resultados:**

incluíram-se 413 estudantes, maioria do sexo feminino, com idade média de 20,5 anos e matriculada em cursos da saúde. Observaram-se vulnerabilidades comportamentais, como não uso de preservativo e uso de álcool/outras drogas, baixa percepção de risco, e conhecimento ruim sobre sífilis. Menos da metade dos estudantes estava disposta a realizar o teste para sífilis.

**Conclusões:**

a adesão ao rastreio para sífilis foi baixa e associada a fatores incomuns, como menor conhecimento, baixa percepção de risco, menor idade e número de parcerias sexuais e frequentar cursos de áreas diferentes da saúde.

## INTRODUÇÃO

As infeções sexualmente transmissíveis (ISTs) mantêm sua marca histórica e, em pleno século XXI, continuam a desafiar a saúde pública globalmente, afetando desproporcionalmente jovens e impactando negativamente a qualidade de vida dos afetados e de suas famílias. Estima-se que, mundialmente, um milhão de novos casos de ISTs ocorram a cada dia e que, desses, 374 milhões sejam de infecções curáveis, como sífilis (7,1 milhões), clamídia (129 milhões), gonorreia (82 milhões) e tricomoníase (156 milhões)^(1)^.

Entre as ISTs, a sífilis tem se destacado no cenário epidemiológico mundial, com aumento de casos na população de jovens adultos. Embora possua terapêutica estabelecida, de baixo custo e simples implementação, a doença apresenta alta morbidade e ocasiona uma série de complicações, podendo evoluir para a cronicidade, além de facilitar a contaminação pelo Vírus da Imunodeficiência Humana (HIV)^(2-4)^.

Em 2016, foram registrados 29.365 casos de sífilis na União Europeia (incidência: 6,1/100.000), e, em 2019, esse número subiu para 35.039 novos casos (incidência: 7,4/100.000). Portugal é um dos países com maior incidência de casos nos últimos anos, com quase 50% das ocorrências na população com idades entre 15 e 35 anos. No país, os casos de sífilis passaram de 43 casos notificados em 2015 (0,4 casos por 100.000 habitantes) para 419 casos em 2019 (4,1 casos por 100.000 habitantes), representando um aumento de 874,42% no número de notificações no país^(4)^.

A vulnerabilidade dos jovens à sífilis e outras ISTs é diversa, envolvendo aspectos como a iniciação sexual precoce, troca frequente de parcerias sexuais e uso irregular do preservativo. Somam-se a esses a necessidade de aceitação e inserção em grupos sociais, e o consumo de álcool e outras drogas^(4-6)^. A entrada no nível superior de ensino parece contribuir com o aumento da vulnerabilidade dos jovens às ISTs. A vida universitária possibilita ao jovem mais autonomia e liberdade para ampliar o seu círculo de amizades e vivenciar novas experiências como uso de álcool, drogas e prática do sexo inseguro, antes limitadas ou inibidas pela maior proximidade e controle familiar^(7)^.

Um dos principais fatores que dificultam o diagnóstico precoce da sífilis é o longo período de latência da infecção. Durante essa fase, em que as pessoas permanecem assintomáticas, geralmente não há motivação para a realização do exame, o que contraria a recomendação da Organização Mundial da Saúde (OMS) de que todas as pessoas expostas realizem testes para ISTs. O diagnóstico precoce tem impacto positivo, pois permite a instituição de tratamento adequado, a redução de transmissão entre parceiros e das complicações em longo prazo, principalmente sobre a saúde reprodutiva dos jovens, além de evitar novos casos de sífilis congênita^(1,8)^.

A prevenção e controle das ISTs, principalmente da infecção pelo HIV, sífilis e hepatites B e C, estão interligados com a promoção da saúde, igualdade de gênero, educação e redução das desigualdades, sendo, portanto, fundamentais para o alcance dos Objetivos de Desenvolvimento Sustentável (ODS) da Agenda 2030 da Organização das Nações Unidas (ONU), que busca responder aos principais desafios globais do século XXI, como a melhoria da saúde individual, saúde sexual e do bem-estar de todas as pessoas^(1)^. Entre as estratégias para ampliar e antecipar o diagnóstico desses infecções, destacam-se o incentivo e a mobilização dos indivíduos para testagem voluntária, principalmente de populações mais vulneráveis, como é o caso dos adolescentes e jovens e, preferencialmente, por meio de testes rápidos (TRs)^(4)^.

Apesar do aumento nos casos notificados de ISTs, há uma escassez de estudos que investigam a epidemiologia dessas infecções em Portugal, especialmente entre os jovens. Dado que o ambiente universitário pode fomentar comportamentos de risco que ampliam a vulnerabilidade dos estudantes, pesquisas que exploram e analisam esses comportamentos se tornam fundamentais para subsidiar a elaboração de políticas públicas e programas de promoção à saúde direcionados a essa população.

## OBJETIVOS

Investigar os fatores associados à disposição de estudantes do ensino superior para conhecer o estado sorológico para sífilis.

## MÉTODOS

### Aspectos éticos

Este estudo é um recorte de investigação de pós-doutoramento intitulada “Perfil epidemiológico, conhecimento e práticas comportamentais relacionadas às infecções de transmissão sexual entre estudantes universitários”. Foi conduzido de acordo com as diretrizes de ética nacionais e internacionais, aprovado pelos Comitês de Ética em Pesquisa da Escola Superior de Enfermagem de Coimbra, em Portugal, e da Universidade Federal de São Paulo, no Brasil.

### Desenho, local e período do estudo

Trata-se de estudo epidemiológico descritivo-correlacional de corte transversal, norteado pelo *checklist* da ferramenta *STrengthening the Reporting of OBservational studies in Epidemiology*. Foi realizado na cidade de Coimbra, região central de Portugal, com dados oriundos de duas instituições de ensino superior, no período de outubro de 2022 a janeiro de 2023.

### Amostra; critérios de inclusão e exclusão

A amostra inicial envolveu 599 estudantes de diversas áreas do conhecimento, selecionados por conveniência. Os critérios para participação incluíam ter idade entre 18 e 25 anos, estar matriculado de forma regular em um dos cursos oferecidos pelas instituições e ter iniciado a vida sexual, resultando em uma amostra final de 413 estudantes.

### Protocolo do estudo

A coleta de dados foi realizada por meio de questionário *online*, enviado aos estudantes via plataforma *Google Forms^®^,* pelas instituições de ensino superior participantes do estudo. O Termo de Consentimento Livre e Esclarecido foi obtido de todos os indivíduos envolvidos no estudo antes do acesso ao questionário.

O instrumento abordava questões sobre os dados sociodemográficos, conhecimentos, comportamentos e atitudes acerca das ISTs, além de uma questão sobre o interesse do estudante em realizar o TR para sífilis, considerada como variável dependente neste recorte. A validação do conteúdo do questionário foi feita por três especialistas da área, sendo dois de Portugal e um do Brasil. Além disso, o instrumento foi submetido a pré-teste com uma turma de 20 estudantes universitários, não incluídos neste estudo.

Para avaliar o conhecimento sobre sífilis, foram realizadas cinco perguntas. A resposta a essas questões foi categorizada em certa e errada/não sabe. O conhecimento foi classificado atribuindo-se “bom” àqueles que responderam corretamente de quatro a cinco perguntas, “regular”, aos que acertaram três, e “ruim”, aos que acertaram de zero a duas questões.

Os dados da pesquisa, gerados em planilha *Microsoft Excel* ^®^ pela plataforma *Google Forms* ^®^, foram transferidos para um banco criado no programa *Statistical Package for the Social Sciences*, versão 24, para Windows, onde as variáveis foram organizadas e, quando necessário, recategorizadas.

### Análise dos resultados e estatística

Para caracterizar a amostra, foi realizada estatística descritiva por meio de distribuição de frequências absolutas e relativas, médias e desvio padrão (DP). A magnitude da associação entre a variável dependente e as demais variáveis investigadas foi estimada por meio da Razão de Prevalência, através do modelo de regressão logística de Poisson com variância robusta.

Inicialmente, foram realizadas análises bivariadas, utilizando-se os testes qui-quadrado de Pearson e Exato de Fisher, quando conveniente, para as variáveis categóricas e, para as variáveis numéricas, o teste t de Student (amostras independentes). Por fim, as variáveis que apresentaram nível descritivo (valor de p em até 0,20) foram selecionadas para o modelo múltiplo, cujo nível de significância adotado foi de 0,05. O modelo multivariável final foi construído utilizando o método *backward* que retira os fatores com valor de p > 0,05 um a um do modelo.

## RESULTADOS

A amostra foi constituída pela maioria de jovens do sexo feminino (79,9%), com idades entre 18 e 25 anos, cor autoclassificada como branca (93,7%), solteira (97,6%), pertencente à religião católica (67,7%) e procedente principalmente da cidade de Coimbra (34,9%). Quanto ao curso frequentado, a área da saúde se destacou (45,3%) em relação às demais áreas, e mais da metade da amostra (53,7%) estava matriculada nos anos finais da sua formação (Tabela 1).

**Tabela 1:** Distribuição das variáveis sociodemográficas e associação com a disposição para o rastreio da sífilis por meio do teste rápido, Coimbra, Portugal, 2022-2023

Variáveis			Adesão ao rastreio de sífilis				Valor de *p*
			Sim		Não		
	n^*^	%	n	%	n	%	
Sexo							
Feminino	330	79,9	97	30,5	221	69,5	0,457
Masculino	83	20,1	21	26,3	59	73,8	
Idade em anos (M±DP)	20,50 ±1,73						
18-19	119	28,8	27	23,5	88	76,5	0,086
20-25	294	71,2	91	32,2	192	67,8	
Cor de pele							
Branca	387	93,7	109	29,3	263	70,7	0,566
Não branca	26	6,3	09	34,6	17	65,4	
Situação conjugal							
Casado/união estável	10	2,4	04	40,0	06	60,0	0,493 (F)
Solteiro	401	97,6	114	29,5	272	70,5	
Procedência							
Coimbra	144	34,9	38	27,5	100	72,5	0,215
Outras cidades de Portugal	255	61,7	73	29,7	173	70,3	
Outros países	14	3,4	07	50,0	07	50,0	
Área do curso							
Saúde	187	45,3	82	45,3	99	54,7	< 0,001
Outras áreas	226	54,7	36	16,6	181	83,4	
Ano do curso							
1º e 2º anos	191	46,2	49	26,6	135	73,4	0,222
3º ano ou mais	222	53,8	69	32,2	145	67,8	

M - média; DP - desvio padrão;

* Valores com somas diferentes de 413 referem-se a missing data.

As variáveis sociodemográficas que se associaram ao desfecho (p< 0,20) e que foram incluídas no modelo de regressão logística de Poisson foram idade e área do curso frequentado (Tabela 1).

Quanto ao conhecimento sobre sífilis (Tabela 2), a média de acertos entre as cinco questões foi de 2,33 (DP = 1,54). A questão com maior frequência de acertos foi “A sífilis não tratada pode causar problemas neurológicos e cardiovasculares” (57,7%), e a com maior frequência de erros foi “Manchas pelo corpo e queda de cabelo podem ser sinais de sífilis” (31,5%).

**Tabela 2:** Conhecimento sobre sífilis entre estudantes do ensino superior, Coimbra, Portugal, 2022-2023

Questões avaliadas	n^*^	%
A sífilis não tratada pode causar problemas neurológicos e cardiovasculares		
Resposta correta	240	58,3
Resposta incorreta/não sabe	172	41,7
A sífilis é uma infecção sexualmente transmissível sem cura, mas que pode ser prevenida por meio de vacina		
Resposta correta	204	49,5
Resposta incorreta/não sabe	208	50,5
A sífilis pode ser transmitida por meio do uso de sanitários, piscinas, bancos de jardins		
Resposta correta	187	45,4
Resposta incorreta/não sabe	225	54,6
Manchas pelo corpo e queda de cabelo podem ser sinais de sífilis		
Resposta correta	136	32,9
Resposta incorreta/não sabe	277	67,1
A sífilis durante a gestação pode causar aborto, parto prematuro e nascimento de crianças com problemas graves de saúde		
Resposta correta	213	51,8
Resposta incorreta/não sabe	198	48,2
Número médio de acertos (M ± DP)	2,33 ±1,54	

M - média; DP - desvio padrão;

* Valores com somas diferentes de 413 referem-se a missing data.

Na classificação geral, prevaleceu o nível ruim de conhecimento acerca das questões avaliadas (Tabela 3). A Tabela 3 apresenta os resultados relacionados às variáveis comportamentais dos estudantes investigados, ao conhecimento sobre sífilis e à associação entre essas variáveis e disposição para realizar o rastreio por meio do TR.

**Tabela 3:** Características comportamentais e conhecimento sobre sífilis de estudantes do ensino superior e associação com a disposição para o rastreio da sífilis por meio do teste rápido, Coimbra, Portugal, 2022-2023

Variáveis			Disposição para realizar o teste rápido para sífilis				Valor de *p*
			Sim		Não		
	n^*^	%	n	%	n	%	
Orientação sexual							
Heterossexual	330	84,2	91	28,5	228	71,5	0,076
Homossexual/bissexual	62	15,8	24	40,0	36	60,0	
Idade da sexarca (M ± DP)	16,73 ± 1,71						
Mais de 18 anos	48	12,6	13	27,1	35	72,9	0,340
16-18 anos	247	64,8	68	28,5	171	71,5	
15 anos ou menos	86	22,6	31	36,5	54	63,5	
Parceiro(a) fixo(a) nos últimos 6 meses							
Não	92	22,3	22	23,9	70	76,1	0,262
Sim	321	77,7	96	29,9	225	70,1	
Parceiro(a) casual nos últimos 6 meses							
Não	231	55,9	60	26,0	171	74	0,188
Sim	182	44,1	58	31,9	124	68,1	
Número de parcerias sexuais fixas^**^ (M ± DP)	0,86 ± 0,56						
Nenhum	62	15,0	15	25,4	44	74,6	0,676
Um(a) parceiro(a)	289	70,0	84	29,9	197	70,1	
Dois(duas) ou mais parceiros(as)	62	15,0	19	32,8	39	67,2	
Nº parcerias sexuais casuais^**^ (M ± DP)	0,90 ± 1,65						
Nenhum	192	51,3	52	28,0	134	72,0	0,437
Um(a) a dois(duas) parceiros(as)	141	37,7	44	32,1	93	67,9	
Três ou mais parceiros(as)	41	11,0	15	37,5	25	62,5	
Nº total de parcerias sexuais^**^ (M ± DP)	0,90 ± 1,65						
Até 2 parceiros(as)	347	84,0	92	26,5	255	73,5	0,034
3 ou mais parceiros(as)	66	16,0	26	39,4	40	60,6	
Uso do preservativo na 1ª relação sexual							
Sim	287	69,5	82	29,7	194	70,3	0,968
Não/não lembra	126	30,5	36	29,5	86	70,5	
Uso do preservativo no sexo vaginal/anal^**^							
Sim/maioria das vezes	171	49,1	47	27,5	124	72,5	0,283
Não/minoria das vezes	177	50,9	58	32,8	119	67,2	
Uso de preservativo no sexo oral^**^							
Sim/maioria das vezes	69	18,2	16	23,2	53	76,8	0,188
Não/minoria das vezes	311	81,8	97	31,2	214	68,8	
História anterior ou atual de infecções sexualmente transmissíveis							
Não/não sabe	387	93,7	112	28,9	275	71,1	0,005
Sim	26	6,3	6	23,1	20	76,9	
Uso de substâncias psicoativas^**^							
Não	34	8,2	15	44,1	19	55,9	0,036
Usa/já usou	379	91,8	103	27,2	276	72,8	
Percebeu-se em risco para contrair infecções sexualmente transmissíveis							
Sim	141	34,1	54	38,3	87	61,7	0,002
Não/não lembra	272	65,9	64	23,5	208	76,5	
Fez exame para sífilis anteriormente							
Sim	40	10,0	15	37,5	25	62,5	0,252
Não/não lembra	358	90,0	103	28,8	255	71,2	
Conhecimento sobre sífilis							
Bom	101	24,5	52	53,1	46	46,9	< 0,001
Regular	91	22,0	25	28,7	62	71,3	
Ruim	221	53,5	41	19,2	172	80,8	

* Valores com somas diferentes de 413 referem-se a missing data;

** Informações referentes aos últimos 6 meses.

A partir da análise bivariada (Tabela 3), foram selecionadas para comporem o modelo de regressão logística de Poisson as seguintes variáveis (p< 0,20): orientação sexual; teve parceiro(a) casual/eventual nos últimos 6 meses; número total de parceiros(as) sexuais nos últimos 6 meses (fixos + casuais); uso de preservativo no sexo oral nos últimos 6 meses; uso de substâncias psicoativas (álcool e ou outras drogas); percebeu-se em risco para contrair IST alguma vez na vida; e conhecimento sobre sífilis.

Quanto às variáveis numéricas, foram selecionadas pelo teste t para amostras independentes aquelas que apresentaram nível de significância de até 0,20, tais como idade em anos (p=0,018), n° de parceiros(as) fixos(as) nos últimos 6 meses (p**=** 0,160) e nº total de parceiros(as) sexuais nos últimos 6 meses (p= 0,151) (dados não apresentados em tabela).

Após ajuste, o modelo logístico final ficou constituído pelas variáveis (p< 0,05): área do curso, conhecimento sobre sífilis, percepção de risco para IST, n° total de parceiros(as) sexuais nos últimos 6 meses e idade em anos (Tabela 4).

**Tabela 4:** Razão de Prevalência das variáveis associadas à adesão ao rastreio da sífilis entre estudantes do ensino superior, Coimbra Portugal, 2022-2023 (estimativas do modelo logístico)

Variáveis independentes	RP ajustada	IC 95%	Valor de *p* ^*^
Área do curso			
Saúde	1		
Outras áreas	1,158	1,094-1,225	<0,001
Conhecimento sobre sífilis			
Bom	1		
Regular	1,152	1,069-1,240	<0,001
Ruim	1,137	1,045-1,236	0,003
Percepção de risco para infecções sexualmente transmissíveis			
Sim	1		
Não	1,069	1,011-1,130	0,019
Nº total de parceiros(as) sexuais nos últimos 6 meses			
Até 2 parceiros(as)	1		
3 ou mais parceiros(as)	0,916	0,850-0,987	0,008
Idade em anos	0,980	0,966-0,995	0,021

RP - Razão de Prevalência; IC - Intervalo de Confiança;

* teste qui-quadrado de Pearson.

## DISCUSSÃO

Em termos gerais, o perfil sociocomportamental dos estudantes do ensino superior encontrado neste estudo se assemelha ao descrito em outros estudos nacionais e internacionais^(9-13)^, ou seja, é em sua maioria composto por estudantes do sexo feminino, jovens, solteiros, que assumem comportamentos mais arriscados, como baixa adesão ao preservativo, uso de álcool e outras drogas.

Essas características são consideradas marcadores de maior vulnerabilidade para ISTs e, quando associadas ao ambiente universitário, colocam os estudantes do nível superior como uma população que precisa de maior atenção e visibilidade nas políticas públicas de saúde^(9,11,14)^.

Considerando a sífilis uma infecção que traz graves consequências à saúde humana, principalmente quando acomete o feto, entende-se que a sua prevenção seja urgente e necessária, especialmente na população em idade reprodutiva. Dessa forma, assim como para outras ISTs, autoridades de saúde mundiais recomendam, para além do uso do preservativo, alternativas de prevenção e, entre elas, a testagem periódica para as ISTs^(1,2,4)^.

Para isso, os TRs são considerados uma boa estratégia, pois fornecem o resultado em até 30 minutos, o que permite que medidas sejam tomadas de forma imediata em caso de resultados reagentes^(2)^. Assim, o TR é mais um exemplo da ação pública, com objetivo de controle das ISTs, que se torna mais eficaz quanto maior for a participação dos cidadãos sob a forma de adesão voluntária ao procedimento^(15)^.

No entanto, estudos^(4-6,10,13,16,17)^ têm demonstrado um baixo interesse e adesão aos rastreios para as ISTs entre os jovens, o que é corroborado pelo presente estudo, no qual menos de 30% dos estudantes manifestaram interesse pela testagem rápida para sífilis. O que leva um indivíduo a aceitar ou rejeitar um convite para fazer um exame de rasteio pode ser determinado por diversos fatores e, entre eles, destaca-se a autopercepção de risco/vulnerabilidade diante da situação, ou agravo em questão.

A percepção do risco subjetivo é uma das dimensões propostas pelo Modelo de Crenças da Saúde (MCS), inicialmente desenvolvido por Rosenstock, nos meados do século XX, dimensão esta designada suscetibilidade percebida, uma das preditoras dos comportamentos preventivos de saúde propostos. A suscetibilidade percebida varia de pessoa para pessoa, e refere-se ao risco subjetivo que a pessoa tem de contrair uma doença^(18)^.

Esse modelo teórico foi comprovado por estudos que identificaram a ausência ou baixa percepção de risco como o principal obstáculo à adesão a comportamentos preventivos, inclusive a realização de testes diagnósticos para ISTs^(3,19,20)^. De fato, neste estudo, uma elevada frequência de estudantes (65,9%) não se percebeu em risco para adquirir ISTs, no entanto, de forma contraditória, a análise multivariada revelou que foram justamente esses estudantes que aderiram, de maneira mais significativa, ao rastreio para a sífilis, possivelmente motivados pela curiosidade.

Esse achado demonstra, assim como prevê o MCS, que a percepção de risco por si só não define qual será a ação do indivíduo e que outras dimensões devem ser consideradas, como: a gravidade percebida, ou seja, a percepção que o indivíduo tem sobre o impacto que o evento poderá ocasionar na sua vida; os benefícios percebidos que tais ações preventivas e mudanças de hábito proporcionarão; e as barreiras, dificuldades ou outros aspectos percebidos como negativos relacionados à ação^(18)^.

Assim, outra hipótese para a baixa adesão ao teste encontrada neste estudo seria a falta de percepção quanto à gravidade da sífilis, o que pode ser inferido a partir do baixo conhecimento dos estudantes investigados em relação à infecção ou ao fato de tratar-se de uma infecção curável, o que reduz a preocupação com a sua transmissão^(3)^.

Por fim, as barreiras percebidas pelo estudante podem superar os benefícios e contribuir para não adesão ao rastreio, mesmo entre aqueles que percebem a sua vulnerabilidade e gravidade da infecção. Sentimentos negativos, vinculados aos estereótipos sobre comportamento e moralidade social que circundam ainda hoje o universo das ISTs, como medo, vergonha e constrangimento, podem melhor explicar a baixa adesão ao rastreio, como já demonstrado por outros estudos^(21-22)^.

Outro achado considerado inesperado neste estudo foi a prevalência de adesão ao rastreio significativamente maior entre os estudantes que tiveram menor número de parcerias sexuais no último semestre. Na verdade, o oposto seria o esperado, visto que trocar de parceria sexual com maior frequência deixa as pessoas mais expostas às ISTs, o que, normalmente, impulsiona a busca pelo teste.

No entanto, nem sempre as pessoas vulneráveis se percebem em risco e, entre as que se percebem, a atitude para não se testar pode estar ancorada na hipótese anterior: dos sentimentos negativos como barreiras para testagem. Além disso, pessoas podem não cogitar realizar testes para ISTs por estarem aparentemente saudáveis^(23)^.

Embora estudos tenham demonstrado associação positiva entre o conhecimento e a intenção ou realização do teste de rastreio para ISTs^(14,23,24)^, no presente estudo, a associação encontrada foi inversa, ou seja, a intenção de realizar o teste foi mais prevalente entre estudantes que apresentaram menor conhecimento sobre sífilis. Também era esperado que os estudantes da área da saúde que, teoricamente, possuem maior conhecimento sobre as ISTs, fossem aderir de forma mais significativa ao rastreio, o que também não ocorreu.

Esses achados corroboram e reforçam a hipótese de que o conhecimento, apesar de fundamental, nem sempre é suficiente para a adoção de medidas preventivas, pois existem fatores intervenientes, relacionados às normas sociais e culturais, pressões exercidas pelo grupo e à própria rede social que podem se sobrepor e levar a condutas inadequadas, observadas neste e em outros estudos^(7,8,14,21)^, como utilização irregular do preservativo, troca frequente de parcerias sexuais, uso de álcool e outras drogas, e baixa realização ou disposição para realizar rastreios diagnósticos.

Por último, verificou-se que a idade influenciou a decisão de realizar o TR para sífilis, demonstrando que a cada um ano de aumento na idade houve diminuição de 2% na prevalência de estudantes com intensão de aderir ao rastreio. Tal resultado se contrapõe ao que é esperado, visto que, à medida que as pessoas amadurecem, tendem a ter maior percepção diante dos riscos já vivenciados e, com isso, maior motivação para realização de rastreios diagnósticos^(17)^.

No entanto, como já discutido, a percepção de risco dos estudantes, no presente estudo, revelou-se um fraco preditor de prevenção, associada de forma negativa à disposição para o rastreio, o que, certamente não explica a atitude desses jovens.

As variáveis definidas pelo modelo múltiplo, que melhor explicaram a disposição para realização do TR para sífilis, neste estudo, são no mínimo surpreendentes e nos levam a refletir sobre como elas estabelecem uma relação entre si e chegam a uma mesma hipótese: de que a percepção de risco, comum nas pessoas com maior número de parcerias sexuais, que possuem maior conhecimento, que frequentam cursos da área da saúde e que possuem mais idade, pode funcionar, ao contrário do que a maioria dos estudos aponta, como uma barreira para a realização de rastreios diagnósticos.

Considerando que a decisão sobre a realização do teste pode ser complexa, acredita-se que promover o aconselhamento profissional sobre os riscos de infecção e a consciência sobre os benefícios da realização do teste poderiam ajudar na decisão de adesão ao rastreio, o que, infelizmente, não foi possível, devido ao método escolhido para a coleta de dados neste estudo. Em estudo que investigou os fatores associados ao diagnóstico tardio do HIV^(20)^, 41,4% dos entrevistados decidiram se testar somente após o aconselhamento do profissional de saúde, demonstrando, assim, a importância do aconselhamento no contexto das ISTs.

### Limitações do estudo

Este trabalho possui algumas limitações, como a utilização de uma amostra selecionada por conveniência e limitada a uma região geográfica portuguesa, o que pode não traduzir linearmente a realidade do país. Além disso, em virtude do delineamento utilizado, não é possível estabelecer uma relação temporal e causal entre a disposição para realizar o TR para sífilis e as demais variáveis analisadas.

### Contribuições para as áreas da educação, saúde e enfermagem

Apesar das limitações, acredita-se que os resultados apresentados possam favorecer a reflexão sobre a necessidade de adaptações curriculares e desenvolvimento de programas/projetos de pesquisa e extensão nas instituições de ensino superior, visando aumentar a literacia em saúde e promover a saúde sexual dos estudantes. Além disso, espera-se que esses achados incentivem a implementação de políticas institucionais abrangentes de saúde estudantil que não só abordem a saúde sexual, mas também outros aspectos relevantes, consolidando o papel das universidades como promotoras de saúde. Neste contexto, destaca-se a importância da inserção do(a) enfermeiro(a), profissional com competência técnica para fortalecer a promoção da saúde dentro das instituições de ensino superior.

## CONCLUSÕES

A adesão ao teste de rastreio da sífilis entre os estudantes foi baixa, e esteve associada a fatores atípicos, como menor nível de conhecimento, baixa percepção de risco, idade mais jovem, menor número de parcerias sexuais e participação em cursos de áreas não relacionadas à saúde.

Esses resultados nos convidam a refletir sobre os diferentes fatores psicossociais como obstáculos à realização de testes para ISTs e sobre a importância de considerá-los nas discussões e no planejamento de intervenções voltadas ao público em questão. Nesse contexto, é fundamental que as instituições de ensino superior se reconheçam como espaços oportunos de promoção da saúde sexual e reprodutiva de seus estudantes, alinhando-se às orientações da OMS e aos ODS.

## Data Availability

*https://doi.org/10.1590/SciELOPreprints.9513.*
